# Synergistic Effects of *Rhizophagus irregularis* and *Trichoderma harzianum* Co-Inoculation on Enhancing Drought Tolerance and Secondary Metabolite Production in Licorice (*Glycyrrhiza uralensis*)

**DOI:** 10.3390/jof11070488

**Published:** 2025-06-27

**Authors:** Kangxu Zhang, Mengyao Sun, Haiyan Feng, Xia Wei, Wei Xie, Wei Fu, Lanping Guo, Xin Zhang, Zhipeng Hao, Baodong Chen

**Affiliations:** 1 State Key Laboratory of Regional and Urban Ecology, Research Center for Eco-Environmental Sciences, Chinese Academy of Sciences, Beijing 100085, China; zhangkangxu0129@126.com (K.Z.); sunmengyao2025@163.com (M.S.); xieweibisheng@yeah.net (W.X.); weifu@rcees.ac.cn (W.F.); xinzhang@rcees.ac.cn (X.Z.); bdchen@rcees.ac.cn (B.C.); 2 University of Chinese Academy of Sciences, Beijing 100049, China; 3 School of Earth Sciences and Resources, China University of Geosciences (Beijing), Beijing 100083, China; haiyan@cugb.edu.cn; 4 Foshan IronMan Environmental Technology Co., Ltd., Foshan 528051, China; 13138202848@163.com; 5 China CAS Key Laboratory of Mountain Ecological Restoration and Bioresource Utilization & Ecological Restoration and Biodiversity Conservation Key Laboratory of Sichuan Province, Chengdu Institute of Biology, Chinese Academy of Sciences, Chengdu 610041, China; 6 State Key Laboratory for Quality Ensurance and Sustainable Use of Dao-di Herbs, National Resource Center for Chinese Materia Medica, China Academy of Chinese Medical Science, Beijing 100700, China; glp01@126.com

**Keywords:** microbial symbiosis, sustainable cultivation, bioactive compound accumulation, biosynthetic genes, nutrient stoichiometry

## Abstract

Drought stress significantly hinders the cultivation of medicinal plants such as licorice (*Glycyrrhiza uralensis*), valued for its bioactive compounds, glycyrrhizin, and liquiritin. This study aims to investigate how co-inoculation with arbuscular mycorrhizal fungus *Rhizophagus irregularis* and *Trichoderma harzianum* can enhance licorice drought tolerance and secondary metabolite production, providing insights for sustainable agriculture in arid regions. The results demonstrate that inoculation with *R. irregularis* significantly improved biomass, drought stress tolerance, and increased glycyrrhizin and liquiritin concentrations by 29.9% and 3.3-fold, respectively, particularly under drought conditions. Co-inoculation with *T. harzianum* further boosted glycyrrhizin yield by 93.7%, indicating a synergistic relationship between the two microbes. The expression of key biosynthetic genes, including squalene synthase (*SQS1*) for glycyrrhizin and chalcone synthase (*CHS*) for liquiritin, was significantly upregulated, enhancing water use efficiency and the biosynthesis of secondary metabolites. Nutrient analysis showed improved phosphorus uptake, alongside reduced root carbon and nitrogen concentrations, leading to greater nutrient utilization efficiency. These findings suggest that co-inoculating *R. irregularis* and *T. harzianum* is a promising approach to improving licorice growth and medicinal quality under drought stress, with broad applications for sustainable crop management.

## 1. Introduction

Licorice (*Glycyrrhiza uralensis* Fisch.), a perennial leguminous plant with medicinal properties, is commonly cultivated in arid and semi-arid regions worldwide. This widespread cultivation is largely attributed to its diverse applications across industries, such as food, cosmetics, and chemicals [1,2]. Its significant commercial potential stems from its ability to produce various bioactive compounds, including triterpenoids such as glycyrrhizin (C_42_H_62_O_16_), which possess antiviral, anti-inflammatory, and immunomodulatory properties [3,4]. Additionally, phenolic compounds such as liquiritin (C_21_H_22_O_9_) exhibit anti-tumor, anti-allergic, and neuroprotective effects [5,6]. The concentrations of these secondary metabolites are crucial indicators of licorice’s medicinal quality. However, the relatively low levels of secondary metabolites in cultivated licorice roots constrained the availability of high-quality licorice, posing a substantial challenge to the licorice cultivation industry [7].

Secondary metabolites contribute to licorice’s medicinal properties and play an essential role in its defense mechanisms [8]. Their synthesis is often triggered by environmental stressors, particularly drought, which significantly affects plant growth and productivity [9]. Glycyrrhizin, with its potent antioxidant properties, is crucial for scavenging reactive oxygen species and mitigating oxidative stress under drought conditions [10]. While drought typically inhibits overall plant growth, it has been shown to enhance the production of certain secondary metabolites, including glycyrrhizin in licorice [11]. Additionally, drought stress induces the expression of key biosynthetic genes, such as those encoding squalene synthase (*SQS*), thereby boosting triterpene production [12].

Drought stress, a predominant abiotic factor in arid and semi-arid regions, not only reduces biomass, but also lowers the accumulation of bioactive compounds, underscoring the need for strategies to mitigate its impact on licorice cultivation [11]. Beneficial microorganisms, particularly arbuscular mycorrhizal (AM) fungi and *Trichoderma* species, emerged as promising tools to enhance plant resilience under drought conditions [13,14]. These microorganisms form symbiotic relationships with plant roots, improving the plant’s ability to endure stressful environments [15].

AM fungi colonize the root cortex through molecular interactions, forming arbuscular structures that enhance the uptake of critical nutrients, particularly phosphorus (P) [16,17]. In exchange for host-derived lipids and carbohydrates, AM fungi improve soil structure, enhance water and nutrient use efficiency, and modulate plant responses to drought by regulating stress-responsive genes, such as plasma membrane intrinsic protein (*PIP*) [18]. *Trichoderma* species, known for their ability to form symbiotic relationships with plants, further strengthen plant defenses by increasing nutrient uptake, stimulating growth hormone synthesis, and improving drought tolerance [19,20]. These attributes make AM fungi and *Trichoderma* key candidates for improving licorice cultivation under drought conditions [13,21].

Moreover, AM fungi have been shown to enhance the accumulation of secondary metabolites, improving the medicinal quality of crops. For example, AM fungi increase sesquiterpene synthesis in *Atractylodes macrocephala* and boost artemisinin content in *Artemisia annua* [22]. In licorice, *Rhizophagus irregularis* enhances phosphorus absorption and upregulates chalcone synthase (*CHS*) genes involved in liquiritin biosynthesis [23]. Similarly, *Trichoderma* inoculation under drought conditions significantly increases root biomass, root length, and the concentration of bioactive compounds such as calycosin-7-O-β-D-glucoside in *Astragalus mongholicus* [24]. While the individual effects of AM fungi and *Trichoderma* on plant growth are well-documented, their potential synergistic impact on bioactive compound production remains underexplored.

Co-inoculation of AM fungi and *Trichoderma* could theoretically offer complementary benefits under drought conditions, with AM fungi improving phosphorus uptake and nutrient acquisition, while *Trichoderma* stimulates root growth and enhances water uptake [23,25]. This combination could lead to increased biomass and improved secondary metabolite production. However, the mechanisms underlying these interactions and their effects on plant physiology, particularly in terms of secondary metabolite production in licorice, are not fully understood.

We hypothesize that the co-inoculation of *R. irregularis* and *T. harzianum* will improve the drought tolerance and enhance the production of secondary metabolites, such as glycyrrhizin and liquiritin, in *G. uralensis*. This will occur through improved nutrient uptake and the regulation of key biosynthetic and stress-responsive genes under drought conditions. Specifically, we predict that the co-inoculation will increase biomass, elevate concentrations of bioactive compounds, and optimize nutrient stoichiometry, especially under drought stress. This study investigates the combined effects of *R. irregularis* and *T. harzianum* on licorice seedlings, focusing on plant growth, glycyrrhizin and liquiritin concentrations, gene expression related to their biosynthesis, and nutrient uptake. Microbial dependency and drought resistance indices were also evaluated to elucidate the roles of these microorganisms. By clarifying how AM fungi and *Trichoderma* enhance drought tolerance and secondary metabolite production, this study aims to provide insights into sustainable medicinal plant cultivation practices.

## 2. Materials and Methods

### 2.1. Plant and Soil Characteristics

Seeds of *Glycyrrhiza uralensis* Fisch. were sourced from the Chinese Materia Medica Resources Center, China Academy of Chinese Medical Sciences, Beijing, China. To facilitate germination, seeds underwent a 30 min treatment with 50% H_2_SO_4_, followed by three rinses with sterilized distilled water [11]. Subsequently, the seeds were surface-sterilized using a 10% H_2_O_2_ solution for 10 min and again rinsed three times with sterilized distilled water. Germination was carried out in Petri dishes lined with double-layered filter paper in darkness at 25 °C for 2–3 days. Uniformly germinated seeds were selected for pot experiments.

The soil used was collected from the Licorice Cultivation Base, China National Traditional Chinese Medicine Co., Wuwei, China (105.9758° E, 39.0592° N). It had a pH of 8.3, effective nitrogen content of 26.0 mg kg^−1^, extractable phosphorus content of 6.8 mg kg^−1^, and effective potassium content of 59.6 mg kg^−1^. Before use, the soil was subjected to air-drying, followed by grinding and sieving through a 2 mm mesh. It was then sterilized using γ-irradiation (25 kGy) at the Institute of Atomic Energy, Chinese Academy of Agricultural Sciences, Beijing, China. To enhance plant growth, a base fertilizer was added, providing 120 mg kg^−1^ of nitrogen (NH_4_NO_3_), 20 mg kg^−1^ of phosphorus (KH_2_PO_4_), and 120 mg kg^−1^ of potassium (K_2_SO_4_) [11].

### 2.2. Microbial Inoculation

The arbuscular mycorrhizal fungus *Rhizophagus irregularis* AH01 used in this study was originally isolated by our research team from rhizosphere soil collected in Anhui Province, China, and has been stored in the China General Microbiological Culture Collection Center (CGMCC), Beijing, China, under the accession number CGMCC12157. *R. irregularis* was maintained via continuous trap culture using sorghum (*Sorghum bicolor*) plants in sterilized sandy soil under greenhouse conditions. The inoculum, containing 67 spores g^−1^ soil along with hyphae and infected root fragments, was applied to the soil. For the control, an equivalent amount of sterilized inoculum was used. Successful *R. irregularis* inoculation was confirmed by trypan blue staining, with inoculated roots exhibiting over 40% colonization, while non-inoculated roots showed no colonization under microscopic examination.

*Trichoderma harzianum* CFCC82908 was obtained as a dry powder formulation from the China Forestry Culture Collection Center (CFCC), Beijing, China. Prior to use, the dry powder was rehydrated and cultured on potato dextrose agar at 25 °C for 7 days. Hyphal tips were then transferred to sterilized potato dextrose broth and incubated at 25 °C with shaking at 120 rpm for another 7 days. The culture was filtered through sterile gauze, and centrifuged twice (3000× *g*, 5 min), and the conidia collected were washed three times with sterile water. The conidial concentration was adjusted to 3 × 10^6^ conidia mL^−1^ [26]. Inoculation of *T. harzianum* was confirmed by culturing on *Trichoderma* selective medium (TSM), which allows for the selective growth of *Trichoderma* species.

### 2.3. Experimental Design and Growth Conditions

The experiment was structured as a three-factor randomized complete block design, incorporating the following treatments: (1) no inoculation with *R. irregularis* (-Ri) and *T. harzianum* (-Th) as the control; (2) inoculation with *R. irregularis* (+Ri); (3) inoculation with *T. harzianum* (+Th); and (4) co-inoculation with both *R. irregularis* and *T. harzianum* (+Ri+Th). Each microbial treatment was applied under two environmental conditions: well-watered (WW) and drought stress (DS). In total, the experiment consisted of 8 treatments, with 5 replications per treatment.

Germinated seeds were sown in pots containing 400 g of soil. Mycorrhizal inoculation was conducted by applying 30 g of *R. irregularis* inoculum to the top 3 cm of soil. After seven days of growth, 40 mL of *T. harzianum* conidia suspension was added, with the control receiving sterile water.

During the pre-drought period, soil moisture was maintained at 16% (~60% of the saturation moisture content). After 90 days, half of the plants in each inoculation group remained under well-watered conditions, while the other half experienced drought stress (11% soil moisture content, ~40% saturation moisture content) for 28 days before harvest [11].

Greenhouse conditions were controlled at 15–25 °C, with a 16 h photoperiod and supplementary lighting (700 µmol m^−2^ s^−1^). Phosphorus-free Hoagland nutrient solution was applied every 14 days.

### 2.4. Parameter Measurements

Plant roots and shoots were harvested separately, washed with deionized water, and a portion of the roots was flash-frozen in liquid nitrogen for RNA extraction. The remaining biomass was oven-dried at 75 °C for 72 h until a constant weight was achieved. Dried root samples were ground for the analysis of nutrients, glycyrrhizin, and liquiritin content.

The drought stress index (STI) was calculated as STI = (Bc × Bs)/Mc^2^, where Bc represents the biomass under control conditions, Bs denotes the biomass under stress conditions, and Mc is the average biomass under control conditions [27].

The microbial dependency of the inoculation treatment was computed following Smith et al. [28]. MD (%) = 100 (value for inoculation treatment plant—mean value for non-inoculated plants)/value for inoculation treatment plant. These equations ensure that positive and negative values for microbial dependency are comparable and symmetrical [28].

To prepare the root powder for analysis, 0.1 g of the sample was extracted with 100 mL of 70% methanol using an ultrasonic bath (250 W, 40 kHz) for 30 min. Afterward, the solution was cooled and filtered through a 0.45 µm membrane. The quantification of glycyrrhizin and liquiritin was performed using high-performance liquid chromatography (HPLC; Agilent-1200, Santa Clara, CA, USA) equipped with a diode-array detector (DAD) detector (G1315D), employing a ZORBAX Eclipse XDB-C18 column (250 mm × 4.6 mm, 5 µm) [11]. Certified reference standards (Batch No. 110731 for glycyrrhizin and Batch No. 11610 for liquiritin), sourced from the National Institute for the Control of Pharmaceutical and Biological Products in China, were used to create standard calibration curves.

Total RNA was isolated from root samples utilizing the RNeasy Plant Mini Kit (Qiagen, Dusseldorf, Germany). Following DNase I treatment (Thermo Fisher Scientific Inc., Waltham, MA, USA), cDNA synthesis was performed using the RevertAid First Strand cDNA Synthesis Kit (Thermo Fisher Scientific Inc., Waltham, MA, USA). Gene expression levels were quantified using real-time PCR on the LightCycler 480II system (Roche Diagnostics GmbH, Penzberg, Germany) with SYBR Green I (TAKARA Biotechnology Co., Ltd., Kusatsu, Japan). Primers for *PIP*, *SQS1*, and *CHS* were designed as follows: *PIP* (AY781788.1): 5′-ATCACCATCTTGACCGTCATGGG-3′, *SQS1* (HM012836.1): 5′-GCACTCGTCATTCAGCAGCTCGAC-3′, and *CHS* (U37840): 5′-AAAGCTCTTGGGCCTTCATCG-3′ [11]. The PCR protocol consisted of 40 cycles, with each cycle involving 5 s at 95 °C, 45 s at 58 °C, and 30 s at 72 °C. For each sample, three technical replicates and five biological replicates were performed. Gene expression data were normalized to Actin2 and analyzed using the 2^−∆∆Ct^ method as described by Pfaffl [29].

Dried root samples were ground at 1600 rpm for 2 min using a GT200 ball mill (Beijing Grinder Co., Beijing, China). Root samples were digested in 10 mL nitric acid and underwent closed-vessel microwave digestion (Mars5, CEM Corp., Matthews, NC, USA). Carbon and nitrogen concentrations were analyzed using an elemental analyzer (Vario MAX, Elementar, Langenselbold, Germany), while phosphorus concentrations were determined through ICP-OES (Prodigy, Teledyne Leeman, Hudson, NH, USA Prodigy) after nitric acid digestion.

### 2.5. Statistical Analysis

The percentage data were subjected to an arcsine transformation [arcsine square-root (X)] to ensure normality. The normality of the data was evaluated using the Shapiro–Wilk test, and Levene’s test was applied to assess the homogeneity of variances. A one-way analysis of variance (ANOVA) followed by Duncan’s multiple-range test (*p* < 0.05) was performed to compare treatment effects, and a three-way ANOVA was conducted to examine the impacts of water regime (WR), mycorrhizal inoculation (Myc), and *Trichoderma inoculation* (Tri), along with their interactions, excluding microbial response variables. All statistical procedures were carried out using SPSS Statistics 24 (IBM Corp., Armonk, NY, USA), with results presented as mean ± standard error.

## 3. Results

### 3.1. Plant Biomass and Microbial Response

**Figure 1:**
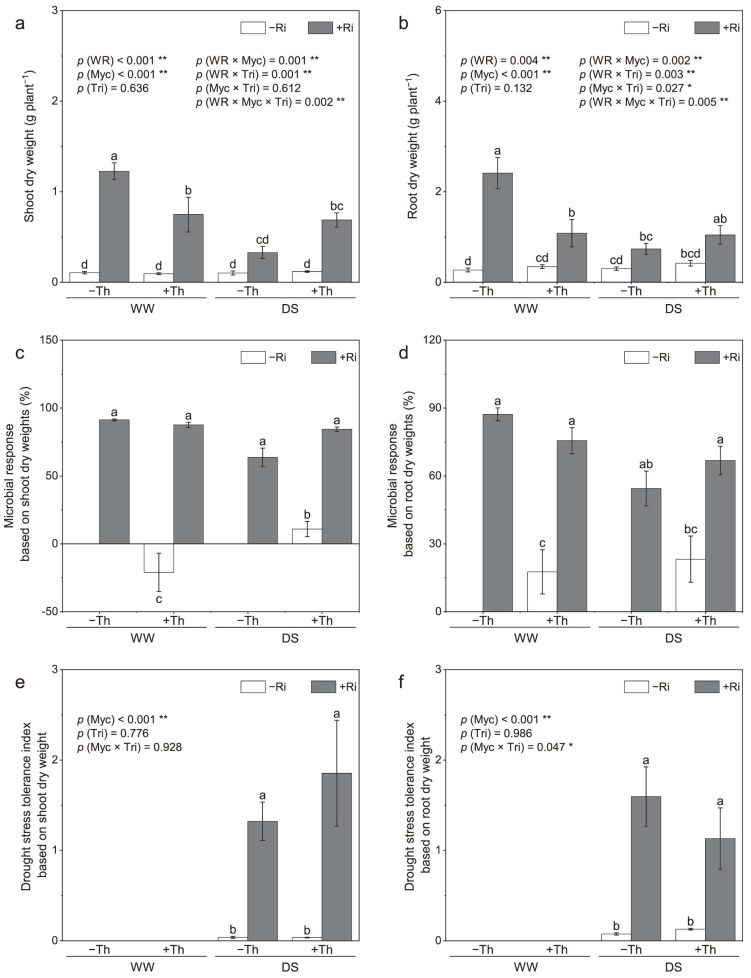
Dry weights of shoots (**a**) and roots (**b**), drought stress tolerance index calculated from shoot (**e**) and root (**f**) dry weights, and microbial response based on shoot (**c**) and root (**d**) dry weights of plants inoculated with or without *Rhizophagus irregularis* (Ri) and *Trichoderma harzianum* (Th) under well-watered (WW) and drought stress (DS) conditions. The labels –Ri and +Ri represent non-inoculated control and inoculation with *R. irregularis*, respectively, and –Th and +Th denote non-inoculated control and inoculation with *T. harzianum*, respectively. WR refers to the water regime. Myc and Tri represent the mycorrhizal and *Trichoderma* inoculation treatments, respectively. Columns with the same lowercase letters indicate no significant difference at *p* < 0.05, with * representing *p* < 0.05 and ** representing *p* < 0.01.

Drought stress resulted in a reduction in plant growth (Figure 1a,b). In the case of AM plants, drought stress significantly lowered both the shoot and root dry weights (*p* < 0.01). A distinct interaction was observed between *R. irregularis* and *T. harzianum* inoculation across different watering conditions (*p* < 0.01). Under drought stress, the combined inoculation of *R. irregularis* and *T. harzianum* resulted in a synergistic effect, promoting shoot and root growth. However, under well-watered conditions, plants inoculated solely with *R. irregularis* showed higher dry weight compared to those co-inoculated with both *R. irregularis* and *T. harzianum*.

Overall, inoculations with both *R. irregularis* and *T. harzianum* had positive effects under various watering conditions, except for a negative impact on shoot growth observed in *T. harzianum* inoculation under well-watered conditions (Figure 1c,d). Co-inoculation with *R. irregularis* and *T. harzianum* led to a significant increase in microbial response under drought stress (*p* < 0.05), but it decreased compared with *R. irregularis* under well-watered conditions. The microbial response of *T. harzianum* increased significantly under drought stress (*p* < 0.05), particularly in terms of shoot growth, indicating a shift from a negative to a positive response.

Inoculation with *R. irregularis* led to a higher shoot STI compared with the non-inoculated control (Figure 1e). Both *R. irregularis* and *T. harzianum* inoculations enhanced the root STI compared to the non-inoculated control, with *R. irregularis* and *T. harzianum* showing increases of about 22.1-fold and 81.6%, respectively (Figure 1f).

### 3.2. Root Glycyrrhizin, Liquiritin Concentrations, and Yields

**Figure 2:**
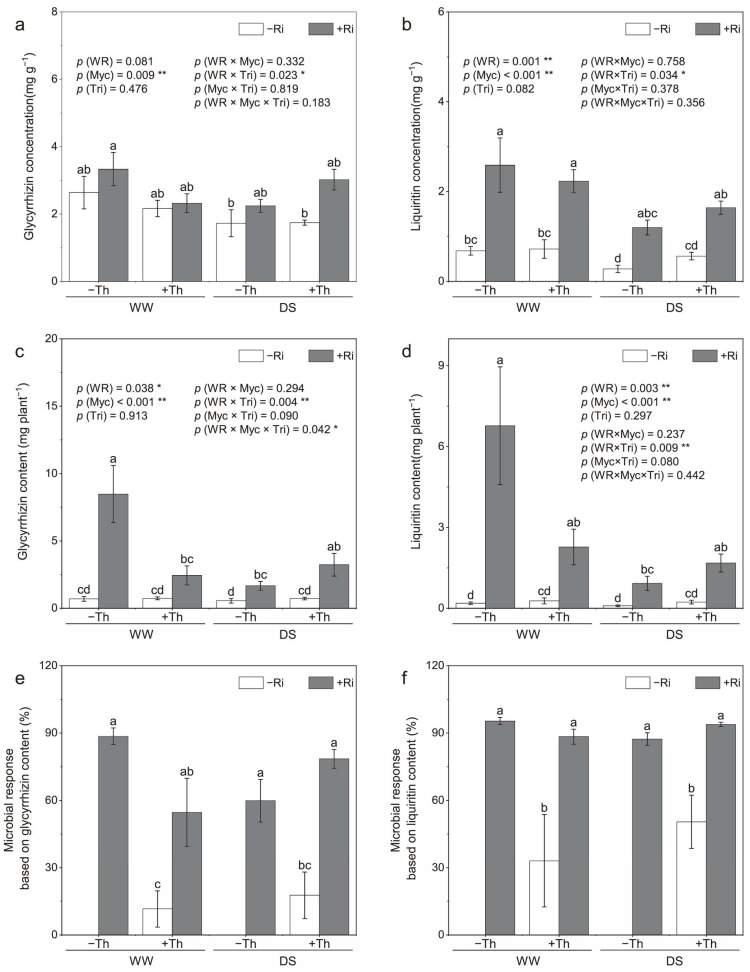
Impact of inoculation with *Rhizophagus irregularis* (Ri) and *Trichoderma harzianum* (Th) on glycyrrhizin concentration (**a**) and content (**c**), liquiritin concentration (**b**) and content (**d**), and microbial response based on glycyrrhizin (**e**) and liquiritin (**f**) content in licorice roots under well-watered (WW) and drought stress (DS) conditions. The labels –Ri and +Ri represent non-inoculated control and inoculation with *R. irregularis*, respectively, and –Th and +Th denote non-inoculated control and inoculation with *T. harzianum*, respectively. WR refers to the water regime. Myc and Tri represent the mycorrhizal and *Trichoderma* inoculation treatments, respectively. Columns with the same lowercase letters indicate no significant difference at *p* < 0.05, with * representing *p* < 0.05 and ** representing *p* < 0.01.

No significant change was observed in the glycyrrhizin concentrations in the roots subjected to drought stress, whereas drought stress significantly decreased the root liquiritin concentrations (*p* < 0.01). Inoculation with *R. irregularis* significantly enhanced the concentrations of both glycyrrhizin (*p* < 0.01) and liquiritin (*p* < 0.001) in the licorice roots. Specifically, under well-watered and drought stress conditions, the glycyrrhizin concentrations increased by 26.4% and 29.9% (Figure 2a), respectively. Similarly, the liquiritin concentrations increased by 2.8-fold and 3.3-fold (Figure 2b) under these conditions. No significant differences were found between *T. harzianum* inoculation and the non-inoculated control in terms of glycyrrhizin and liquiritin concentrations. However, a significant interaction between *T. harzianum* inoculation and water conditions was observed (*p* < 0.05) under well-watered conditions, and *T. harzianum* inoculation decreased glycyrrhizin concentrations by 47.2%.

Drought stress significantly reduced the yields of glycyrrhizin and liquiritin, while the inoculation of *R. irregularis* increased them. Under well-watered and drought stress conditions, *R. irregularis* inoculation increased glycyrrhizin yields by 11.1-fold and 2.0-fold (Figure 2c). Similarly, liquiritin yields increased by 35.0-fold and 9.2-fold (Figure 2d) under these conditions. Although inoculation with *T. harzianum* did not show a main effect on glycyrrhizin and liquiritin yields across conditions, a significant interaction with water regime was observed, indicating that *T. harzianum* contributed to yield improvement, specifically under drought stress (*p* < 0.05). Synergistic effects on glycyrrhizin yields were observed with the co-inoculation of *R. irregularis* and *T. harzianum* under both water conditions. Co-inoculation with *R. irregularis* and *T. harzianum* resulted in a 93.7% increase in glycyrrhizin yields under drought stress, whereas it decreased by 71.2% compared with *R. irregularis* under well-watered conditions.

A positive contribution of *R. irregularis* and *T. harzianum* inoculation was observed for glycyrrhizin and liquiritin yields under both water conditions (Figure 2e,f). Under drought stress, the microbial response of *T. harzianum* significantly increased compared with well-watered conditions. The microbial response from the co-inoculation of *R. irregularis* and *T. harzianum* significantly improved under drought stress, but decreased under well-watered conditions when compared to *R. irregularis* inoculation alone.

### 3.3. Root PIP, SQS1, and CHS Relative Expression

**Figure 3:**
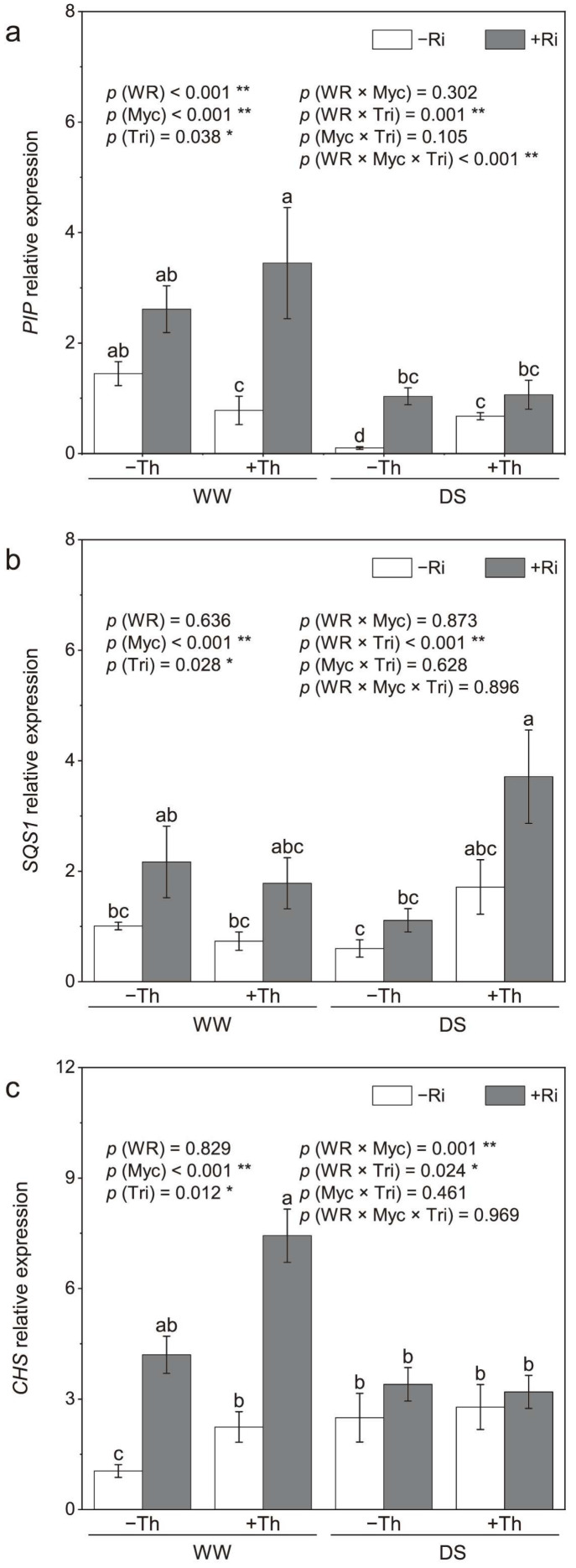
Effects of inoculation with *Rhizophagus irregularis* (Ri) and *Trichoderma harzianum* (Th) on the relative expression of plasma membrane intrinsic protein (*PIP*) (**a**), squalene synthase (*SQS1*) (**b**), and chalcone synthase (*CHS*) (**c**) genes in roots under well-watered (WW) and drought stress (DS) conditions. The labels –Ri and +Ri represent non-inoculated control and inoculation with *R. irregularis*, respectively, and –Th and +Th denote non-inoculated control and inoculation with *T. harzianum*, respectively. WR refers to the water regime. Myc and Tri represent the mycorrhizal and *Trichoderma* inoculation treatments, respectively. Columns with the same lowercase letters indicate no significant difference at *p* < 0.05, with * representing *p* < 0.05 and ** representing *p* < 0.01.

The expression of the *PIP* gene was significantly downregulated under drought stress, but significantly upregulated following inoculation with *R. irregularis* (Figure 3a). *PIP* expression was significantly downregulated by *T. harzianum* inoculation under well-watered conditions, but upregulated under drought stress. Synergistic effects on *PIP* expression were observed with the co-inoculation of *R. irregularis* and *T. harzianum* under both water conditions. Under well-watered conditions, co-inoculation resulted in higher *PIP* expression compared with sole inoculation.

Expression of the *SQS1* was not significantly affected by drought stress (Figure 3b). However, inoculations with *R. irregularis* and *T. harzianum* upregulated *SQS1* expression. Co-inoculation exhibited the highest gene expression under drought stress. The interaction between *T. harzianum* inoculation and water regimes was statistically significant. *SQS1* expression with *T. harzianum* inoculation was 1.9-fold higher under drought stress and 27.1% lower under well-watered conditions compared with the non-inoculated control.

Inoculations with *R. irregularis* and *T. harzianum* significantly upregulated *CHS* expressions, which were unaffected by water regimes (Figure 3c). The interactions of *R. irregularis* inoculation with water regimes and *T. harzianum* inoculation with water regimes were both statistically significant. *CHS* expression with *R. irregularis* inoculation was 36.4% higher under drought stress and 3.0-fold higher under well-watered conditions compared with the non-inoculated control. With *T. harzianum* inoculation, *CHS* expression was 11.6% higher under drought stress and 1.1-fold higher under well-watered conditions compared with the non-inoculated control.

### 3.4. Root Nutrient Concentrations and Stoichiometry

**Figure 4:**
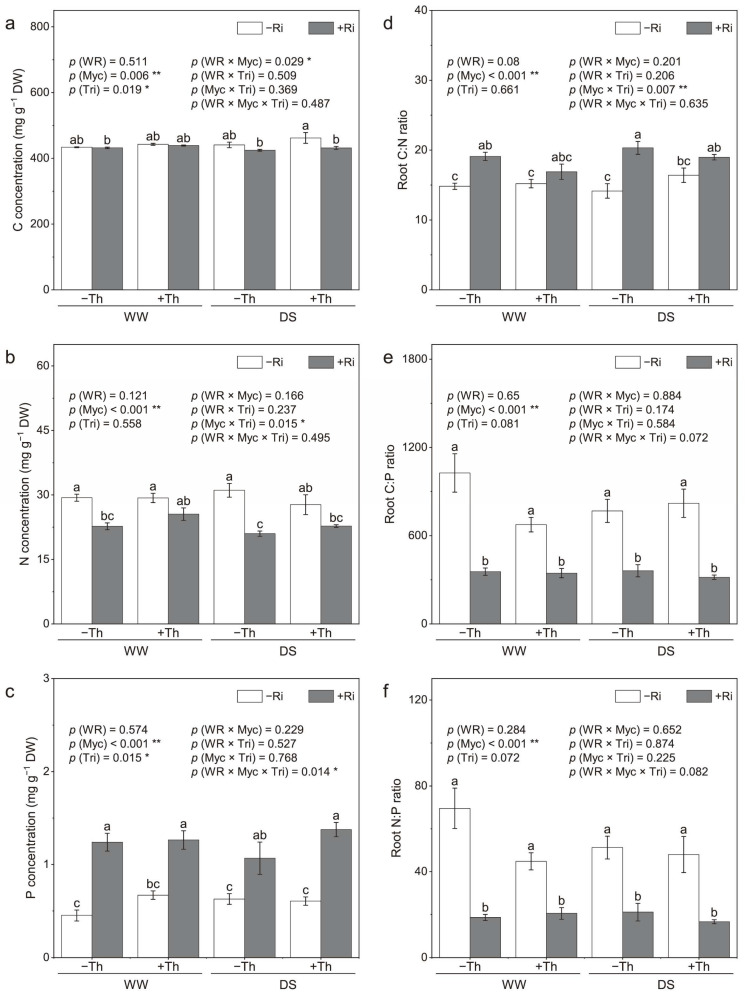
Effects of inoculation with *Rhizophagus irregularis* (Ri) and *Trichoderma harzianum* (Th) on root carbon (**a**), nitrogen (**b**), and phosphorus (**c**) concentrations, as well as C:N (**d**), C:P (**e**), and N:P (**f**) ratios under well-watered (WW) and drought stress (DS) conditions. The labels –Ri and +Ri represent non-inoculated control and inoculation with *R. irregularis*, respectively, and –Th and +Th denote non-inoculated control and inoculation with *T. harzianum*, respectively. WR refers to the water regime. Myc and Tri represent the mycorrhizal and *Trichoderma* inoculation treatments, respectively. Columns with the same lowercase letters indicate no significant difference at *p* < 0.05, with * representing *p* < 0.05 and ** representing *p* < 0.01.

Drought stress did not significantly affect the concentrations of carbon (C), nitrogen (N), or phosphorus (P) in the roots (Figure 4a–c). Inoculation with *R. irregularis* led to a reduction in root C and N concentrations but increased the root P concentration. Conversely, *T. harzianum* inoculation resulted in higher root C and P concentrations. Notably, co-inoculation with *R. irregularis* and *T. harzianum* demonstrated synergistic effects, significantly enhancing root P concentrations under both well-watered and drought stress conditions. Significant interactions between the inoculations of *R. irregularis* and *T. harzianum* were observed in terms of root N concentrations. Co-inoculation increased N concentration by 12.5% and 8.5% under well-watered and drought conditions, respectively, compared to *R. irregularis* inoculation alone.

No significant changes in nutrient stoichiometry were found between the water regimes and *T. harzianum* inoculation (Figure 4d–f). In contrast, *R. irregularis* inoculation significantly altered the root C:N, C:P, and N:P ratios. The C:N ratio increased with *R. irregularis* inoculation, while the C:P and N:P ratios decreased markedly. Significant interactions between *R. irregularis* and *T. harzianum* inoculations were observed specifically in the C:N ratio. Co-inoculation with *R. irregularis* and *T. harzianum* reduced the C:N ratio by 11.6% and 6.6% under well-watered and drought stress conditions, respectively, compared to *R. irregularis* inoculation alone (*p* < 0.05).

### 3.5. Correlations Between Plant Traits

**Figure 5:**
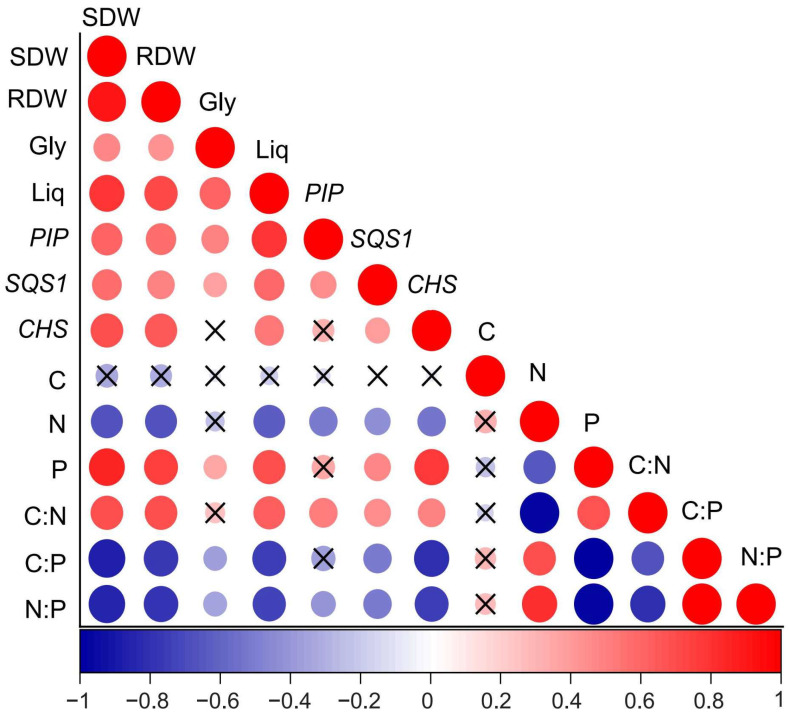
Pairwise correlations of plant traits measured during the study, represented by a color gradient indicating Spearman’s correlation coefficient. The size and color intensity of the circles reflect the strength of the correlation, with larger circles representing stronger correlations. The color scale indicates the nature of the correlation, where a value of 1 represents a perfect positive correlation (shown in red), and -1 indicates a perfect negative correlation (shown in blue). SDW and RDW represent shoot and root dry weights, respectively, while Gly and Liq refer to glycyrrhizin and liquiritin concentrations. *PIP*, *SQS1*, and *CHS* represent the relative expression levels of *PIP*, *SQS1*, and *CHS* genes. “×” denotes no significant difference at *p* < 0.05.

A significant negative correlation was observed between shoot dry weight and root N content, as well as the C:P and N:P ratios, while a positive correlation was found between root P content and the C:N ratio (Figure 5). A similar pattern was observed for root dry weight, which showed negative correlations with root N content, C:P ratio, and N:P ratio, and a positive correlation with root P content and C:N ratio. Additionally, a significant positive correlation was found between glycyrrhizin concentration and the expression of *SQS1*. *CHS* expression also showed a positive correlation with liquiritin concentration. Glycyrrhizin concentration was negatively correlated with both the root C:P and N:P ratios, but positively correlated with root P content. Liquiritin concentration exhibited a similar trend to glycyrrhizin, with negative correlations to root N content, C:P ratio, and N:P ratio, and a positive correlation with root P content and C:N ratio.

## 4. Discussion

This study investigates the synergistic effects of *R. irregularis* and *T. harzianum* on the growth, drought tolerance, and secondary metabolite production of licorice under drought stress. Licorice, known for its valuable medicinal properties, particularly its bioactive compounds glycyrrhizin and liquiritin, often encounters significant challenges in cultivation due to environmental stressors such as drought. This study addresses a crucial gap in understanding how beneficial microorganisms such as AM fungi and *Trichoderma* can mitigate drought stress, enhance plant growth, and promote the production of these key metabolites.

### 4.1. Enhanced Biomass and Stress Tolerance

Drought stress significantly affects plant physiological metabolism, inhibiting growth and reducing biomass. In our study, drought stress indeed reduced licorice growth; however, co-inoculation with *R. irregularis* and *T. harzianum* significantly improved plant growth and enhanced biomass under these stressful conditions. These results align with earlier findings that highlight the positive effects of mycorrhizal fungi on biomass production in stressed environments [8,30]. *R. irregularis* inoculation significantly increased both shoot and root dry weights, particularly under drought conditions, supporting previous research on the role of AM fungi in improving water and nutrient uptake [31,32]. Both AM fungi and *T. harzianum* significantly increased the STI of plant roots compared to the non-inoculated control. The co-inoculation of *R. irregularis* and *T. harzianum* demonstrated synergistic effects, enhancing both biomass and STI, suggesting that microbial inoculation offers an effective strategy for improving plant resilience in arid regions. The increased STI in inoculated plants further underscores the potential of microbial inoculation to mitigate the negative impacts of drought, as shown in other crop studies [33,34].

### 4.2. Accumulation of Secondary Metabolites

Drought stress typically reduces overall biomass but often stimulates secondary metabolite production, which acts as a defense mechanism in plants. Our study observed a significant increase in glycyrrhizin and liquiritin concentrations in *R. irregularis*-inoculated plants under drought stress, with co-inoculation further enhancing these levels. The upregulation of key biosynthetic genes, such as *SQS1* and *CHS*, supports these findings, indicating that microbial inoculation can activate pathways responsible for secondary metabolite production [35]. Mycorrhizal fungi optimize the accumulation of secondary metabolites by enhancing the uptake of essential nutrients such as phosphorus, which is a critical precursor in the biosynthesis of bioactive compounds [23]. Furthermore, AM fungi modulate the expression of biosynthetic genes, thereby improving the plant’s ability to produce bioactive metabolites and enhancing the synthesis of key secondary metabolites [11]. These results are consistent with previous research showing that AM fungal inoculation enhances the concentration of bioactive compounds in medicinal plants [11]. Notably, while *T. harzianum* alone did not significantly affect glycyrrhizin concentration, it contributed to increased liquiritin yield under drought conditions, highlighting its role in modulating specific metabolic pathways [13]. This suggests that dual inoculation offers complementary benefits, improving both plant growth and the medicinal quality of licorice under drought stress [30].

### 4.3. Gene Expression and Stress Response

The significant upregulation of *PIP*, *SQS1*, and *CHS* genes in co-inoculated plants under drought conditions provides insight into the molecular mechanisms driving the observed improvements in drought tolerance and secondary metabolite production. Previous studies established that *PIP* plays a crucial role in regulating water transport under stress conditions [36]. The upregulation of *PIP* in co-inoculated plants indicates enhanced water use efficiency, which is essential for maintaining plant function during drought. SQS1 catalyzes the synthesis of squalene, a key precursor in the triterpene biosynthesis pathway, while CHS is the initial rate-limiting enzyme in the liquiritin biosynthetic pathway [37]. Several studies demonstrated a robust correlation between the expression of these genes and the corresponding concentrations of glycyrrhizin and liquiritin. The upregulation of *SQS1* and *CHS* in this study suggests that microbial inoculation not only improves nutrient uptake, but also activates key metabolic pathways for secondary metabolite synthesis [38]. These findings align with previous studies showing that microbial inoculation can modulate gene expression to enhance plant stress tolerance and metabolite production [11].

### 4.4. Nutrient Utilization and Stoichiometry

Microbial inoculation significantly improves plant mineral nutrition, promotes growth, and enhances resilience to drought stress [39]. In this study, *R. irregularis* and *T. harzianum* inoculation notably affected plant root P concentration, with a significant interaction between water regimes. *R. irregularis* inoculation also led to substantial reductions in the C:N ratios in plant roots, indicating improved nutrient acquisition efficiency, particularly in phosphorus uptake—which is critical for plants under drought conditions [40,41]. The reduction in C:P ratio in co-inoculated plants suggests enhanced nutrient assimilation and utilization, which may explain the observed increases in biomass and secondary metabolite production [42,43]. These changes in nutrient stoichiometry are consistent with research demonstrating that microbial symbiosis can optimize nutrient uptake and distribution, particularly under stress conditions [44].

The co-inoculation of *R. irregularis* and *T. harzianum* showed significant potential for improving drought resistance and secondary metabolite production in licorice, with potential applications for other medicinal plants. This study suggests that microbial inoculation could be integrated into sustainable agricultural practices to enhance crop resilience in arid and semi-arid regions. Given the beneficial effects observed, future research should explore the long-term impacts of co-inoculation on soil health, microbial communities, and plant–microbe interactions across different environmental conditions [45,46]. Further studies are also needed to elucidate the molecular mechanisms underlying these interactions, particularly how AM fungi and *T. harzianum* modulate stress-responsive gene expression and nutrient uptake under varying conditions.

## 5. Conclusions

This study provides new insights into how co-inoculation with *R. irregularis* and *T. harzianum* enhances drought tolerance and secondary metabolite production in licorice. The significant increases in biomass, stress tolerance, and secondary metabolite yields observed in co-inoculated plants highlight the potential of dual microbial inoculation as a sustainable strategy for improving the cultivation of medicinal plants under drought conditions. These findings have important implications for agricultural practices in water-limited environments and offer promising directions for further research into microbial–plant interactions and their application in enhancing crop productivity.

## Data Availability

The original contributions presented in this study are included in the article. Further inquiries can be directed to the corresponding author.
